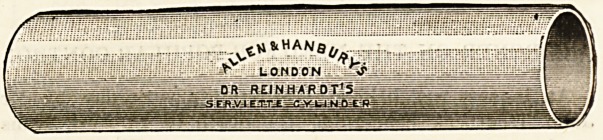# Practical Departments

**Published:** 1903-02-14

**Authors:** 


### PRACTICAL DEPARTMENTS.

### THE SERVIETTE QUESTION IN OPEN-AIR SANATORIA.

By CHARLES REINHARDT, M.D.Brux., L.S.A.Lond.

EVERYONE who has experience of sanatorium life is familiar with the table-napkin question.

At many open-air institutions the patients are provided with Japanese paper serviettes, but these are as a rule satisfactory neither to the patients nor to the members of the staff. In theory they should be a great boon, for they are always clean and are destroyed by burning after being used once, but in practice there are objections.

Nine out of ten patients object to the sensation of applying the paper napkin to their mouths and the tenth does not like its smell, and nearly all would rather be deprived of serviettes altogether than be dependent upon the paper variety. Then they are so light and flimsy that the pleasant breezes which pervade the dining-halls of sanatoria are apt to waft them either off the table altogether or into a neighbour's plate; occasionally they fly out of the dining-room and hide themselves in the shrubs or mingle with the flower-beds. When neatly folded on the table before the meal commences they look attractive if not positively appetising, but when clinging to the branches of a rose-tree, or reposing amongst a bed of pansies, they are not decorative.

There are objections to the use of the linen napkin in that being used for more than one meal and packed away in a drawer in the interval with only the protection of the napkin ring, an undesirable contact between the serviettes of different patients is unavoidable. The phthisical patient who not only uses his table napkin in the ordinary manner, but instinctively raises it to his mouth when obliged to cough at table, cannot fail to infect it with tubercular bacilli; and since the aim of every sanatorium superintendent must be to achieve the most perfect possible condition of hygiene in his institution, he cannot afford to risk infection by serviette.

Various devices are employed at different sanatoria to prevent the contact of table napkins; some of them are not æsthetically satisfactory. At one sanatorium in England washable linen bags are provided for each patient, and though they may answer their purpose they do not look attractive, which is a serious disadvantage, for the table appointments should be entirely unobjectionable in an institution which depends for its success largely upon the work done by the cook in the kitchen and by patients in the dining-room.

To meet the difficulty which ensued when I had to admit that the paper napkin, and the unenclosed linen serviette were unsatisfactory, I devised a serviette cylinder made of solid celluloid with smooth surfaces which is easily cleansed, and which entirely envelopes the napkin, thus protecting it from all contact with others.

These cylinders are not unattractive in appearance, and they are so much appreciated by patients that many ask to be allowed to take one away when leaving the sanatorium. It would be a decided advantage to visitors at boarding-houses where, unfortunately, so many tubercular patients are wont to congregate, were some such arrangement adopted to prevent infection by means of tubercular table napkins, and in families where some member is phthisical the use of the cylinder would be equally advantageous.

At my suggestion, Messrs. Allen and Hanbury have made a supply of serviette cylinders, an illustration of which is reproduced here. They are quite inexpensive.

## Figures and Tables

**Figure f1:**